# Prevention of long-term memory loss after retrieval by an endogenous CaMKII inhibitor

**DOI:** 10.1038/s41598-017-04355-8

**Published:** 2017-06-22

**Authors:** Fabio Antonio Vigil, Keiko Mizuno, Walter Lucchesi, Victoria Valls-Comamala, Karl Peter Giese

**Affiliations:** Department of Basic and Clinical Neuroscience, King’s College London, 125 Coldharbour Lane, London, SE5 9NU United Kingdom

## Abstract

CaMK2N1 and CaMK2N2 are endogenous inhibitors of calcium/calmodulin-dependent protein kinase II (CaMKII), a key synaptic signaling molecule for learning and memory. Here, we investigated the learning and memory function of CaMK2N1 by knocking-down its expression in dorsal hippocampus of mice. We found that reduced CaMK2N1 expression does not affect contextual fear long-term memory (LTM) formation. However, we show that it impairs maintenance of established LTM, but only if retrieval occurs. CaMK2N1 knockdown prevents a decrease of threonine-286 (T286) autophosphorylation of αCaMKII and increases GluA1 levels in hippocampal synapses after retrieval of contextual fear LTM. CaMK2N1 knockdown can also increase CaMK2N2 expression, but we show that such increased expression does not affect LTM after retrieval. We also found that substantial overexpression of CaMK2N2 in dorsal hippocampus impairs LTM formation, but not LTM maintenance, suggesting that CaMKII activity is not required for LTM storage. Taken together, we propose a specific function for CaMK2N1; enabling LTM maintenance after retrieval by inhibiting T286 autophosphorylation of αCaMKII.

## Introduction

CaMKII is a key synaptic signaling molecule that enables learning and memory processes. In particular, the autophosphorylation at T286 of αCaMKII, the major CaMKII isoform in forebrain, is fundamentally important for LTM formation^1–4^. This autophosphorylation not only prolongs αCaMKII activity, it also regulates interaction with various proteins resulting in different downstream effects^5^. For example, a phosphomimetic mutation of the T286 autophosphorylation site increases the immobilization of α-amino-3-hydroxy-5-methyl-4-isoxazolepropionic acid (AMPA) receptors containing the GluA1 subunit within the synapse^6^. The regulation of AMPA receptor localization by CaMKII has been hypothesized to be an important molecular basis for LTM maintenance^7^. However, a role for CaMKII in LTM maintenance is still a matter of debate. Irvine, *et al*.^2^ reported that knock-in mutants lacking the T286 autophosphorylation of αCaMKII form and maintain LTM after intensive training. Additionally, Buard, *et al*.^4^ observed that inhibition of CaMKII, by the CaMK2N1-derived peptide tatCN21, impairs contextual fear LTM formation with no effect on LTM maintenance.

While the biochemistry underlying T286 autophosphorylation of αCaMKII is well known^8^, the regulation of this autophosphorylation is not sufficiently understood. We hypothesized that the endogenous CaMKII inhibitor proteins, CaMK2N1 and CaMK2N2^9, 10^, are important regulators of memory-induced changes in T286 autophosphorylation, as their expression levels are elevated during consolidation of contextual fear memory^11, 12^. CaMK2N1 mRNA levels are increased in the hippocampus after contextual fear conditioning (CFC)^11^. CFC also increases amygdalar CaMK2N2 mRNA expression while hippocampal levels were changed by exposure to a novel context^12^. CaMK2N1 is a 78 amino acid protein with high levels of expression in the frontal cortex, hippocampus and inferior colliculus. CaMK2N2 has 79 amino acids, and is expressed in testis and brain, where it has higher expression in cerebellum and hindbrain. Both inhibitors block CaMKII activity. However, CaMK2N2 but not CaMK2N1 requires constant presence of calcium/calmodulin to bind to CaMKII^10^. The inhibitory effects of both endogenous CaMKII inhibitors seem to be related to the binding of these peptides to a hydrophobic region of the kinase domain of CaMKII, known as the T-site, which blocks access of the substrate to the kinase domain but does not directly block the T286 site. The endogenous inhibitors of CaMKII are also thought to affect how CaMKII interacts with other proteins, regulate CaMKII autophosphorylation at sites other than T286, and decrease calcium/calmodulin dissociation from CaMKII^13^. Despite their regulation during memory consolidation, the importance of CaMK2N1 and CaMKN2 for learning and memory is still unknown. It also remains to be determined if CaMK2N1 and CaKM2N2 have different functions. Here, we have studied the role of CaMK2N1 and, surprisingly, show that this endogenous CaMKII inhibitor is required for maintenance of established LTM after retrieval.

## Results

### CaMK2N1 is not necessary for contextual fear LTM formation, maintenance and retrieval

In order to determine the function of CaMK2N1 in LTM formation and maintenance we prepared an shRNA-expressing recombinant adeno-associated virus (rAAV) to knockdown CaMK2N1 expression. This rAAV and a shRNA-control rAAV were injected into dorsal hippocampi of mice and LTM formation and maintenance were tested in the CFC paradigm. Injection of rAAV in the hippocampi of mice and efficient infection of neurons was confirmed by visualization of ZsGreen reporter protein expression (see Supplementary Fig. S1). The rAAV expressing CaMK2N1 shRNA reduced CaMK2N1 mRNA expression by about 80% in dorsal hippocampus, but did not alter expression levels in ventral hippocampus (Supplementary Fig. S2). For this reason, only dorsal hippocampus samples were used in all following experiments.

**Figure 1:**
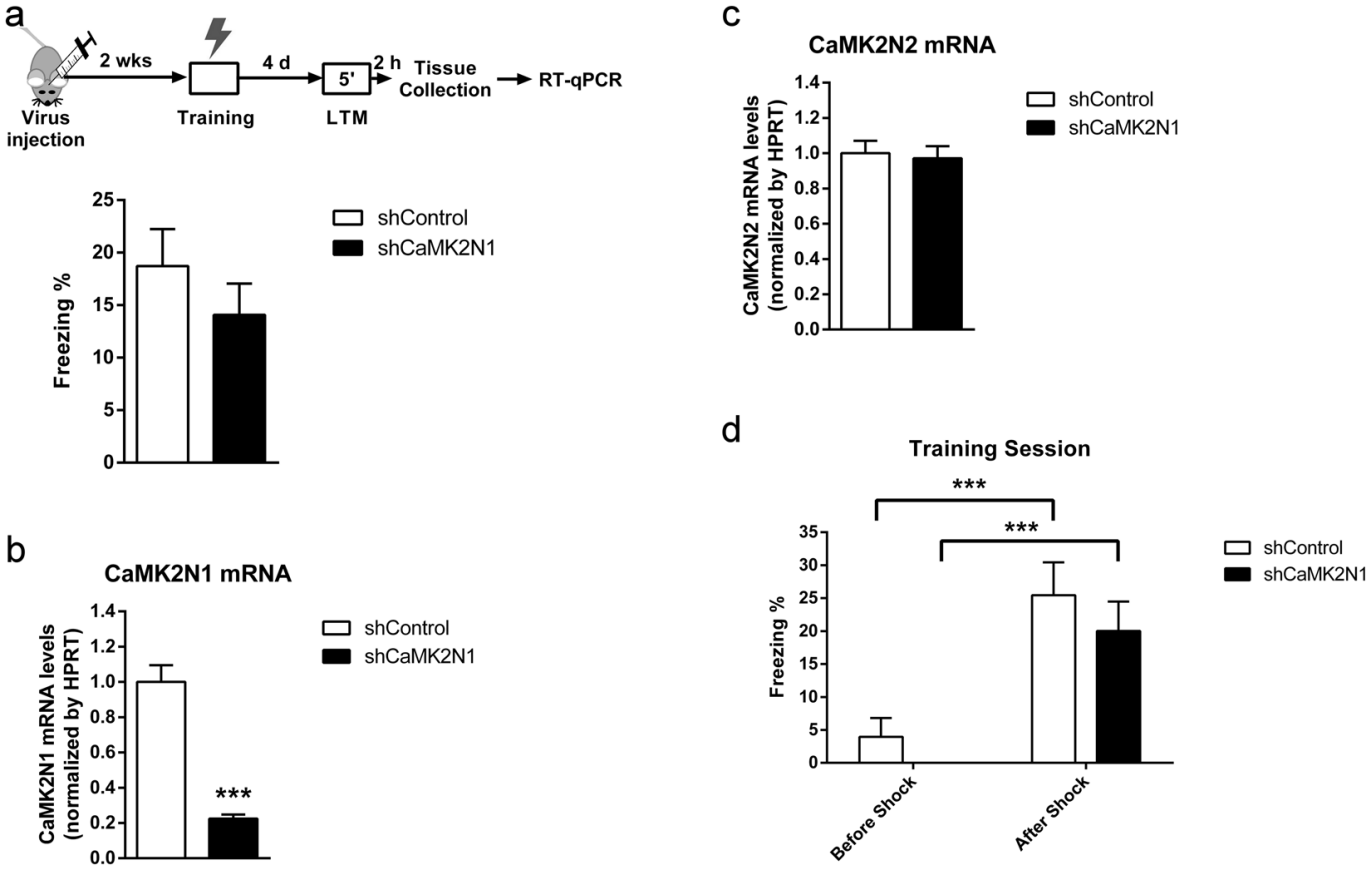
CaMK2N1 shRNA expression in dorsal hippocampus does not affect contextual fear LTM formation, storage and retrieval. (**a**) Dorsal hippocampi of mice were transfected with rAAV expressing either control shRNA (n = 19) or CaMK2N1 shRNA (n = 20). Contextual fear LTM was determined 4 days after conditioning. (**b**) Quantitative analysis of CaMK2N1 mRNA expression in dorsal hippocampi of animals from (**a**) (shControl, n = 19; shCaMK2N1, n = 20). (**c**) Quantitative analysis of CaMK2N2 mRNA expression in dorsal hippocampi of animals from (**a**). (shControl, n = 19; shCaMK2N1, n = 20). (**d**) Freezing scores obtained during conditioning of the animals from (**a**). Training session is divided here between before and after the shock. No animal in the shCaMK2N1 group presented any freezing before shock, resulting in a mean freezing of 0 and 0 of S.E.M. Two-way ANOVA test with repeated measures revealed a significant effect of the foot-shock (F_1,37_ = 32.0; P < 0.001), with no effect of rAAV treatment (F_1,37_ = 1.7; P = 0.120) or interaction between these two factors (F_1,37_ = 0.0; P = 0.84). SNK *post-hoc* comparisons revealed a significant increase in freezing scores between before and after the shock for both shControl (n = 19) (q = 5.7; P < 0.001) and shCaMK2N1 (n = 20) (q = 5.5; P < 0.001) groups. No significant difference was obtained when comparing animals from shControl and shCaMK2N1 within before (q = 1.0; P = 0.44) or after (q = 1.4; P = 0.29) foot-shock. Mean and S.E.M.; ***P < 0.001.

We proceeded to test whether knockdown of CaMK2N1 expression affects contextual fear LTM formation, since CaMK2N1 expression is upregulated in hippocampus during consolidation of this type of memory^11^. Two weeks after transfection with the rAAVs the mice were conditioned and tested for contextual fear LTM after 4 days (Fig. 1a). This experiment was designed to test if the training-induced expression of CaMK2N1^11^ is necessary for learning, consolidation and/or memory retrieval. One-way analysis of variance (ANOVA) of the freezing scores, which are indicative of fear memory, revealed that contextual fear LTM did not differ between the groups (F_1,38_ = 1.0; P = 0.32). This finding shows that reducing CaMK2N1 expression in dorsal hippocampus does not impair contextual fear LTM formation, maintenance and retrieval. The efficient knockdown of CaMK2N1 expression in the dorsal hippocampi of the animals was confirmed by quantitative real-time polymerase chain reaction (RT-qPCR) (F_1,38_ = 66.1; P < 0.001) (Fig. 1b). Specificity of rAVV treatment was confirmed by quantification of CaMK2N2 mRNA levels (F_1,38_ = 0.08; P = 0.77) (Fig. 1c). Freezing scores during CFC training session confirmed that freezing behavior was present only after pairing of shock and context and that shCaMK2N1 treatment did not affect shock reactivity (Fig. 1d).

### Upregulation of CaMK2N1 and CaMK2N2 mRNA after contextual fear LTM retrieval

**Figure 2:**
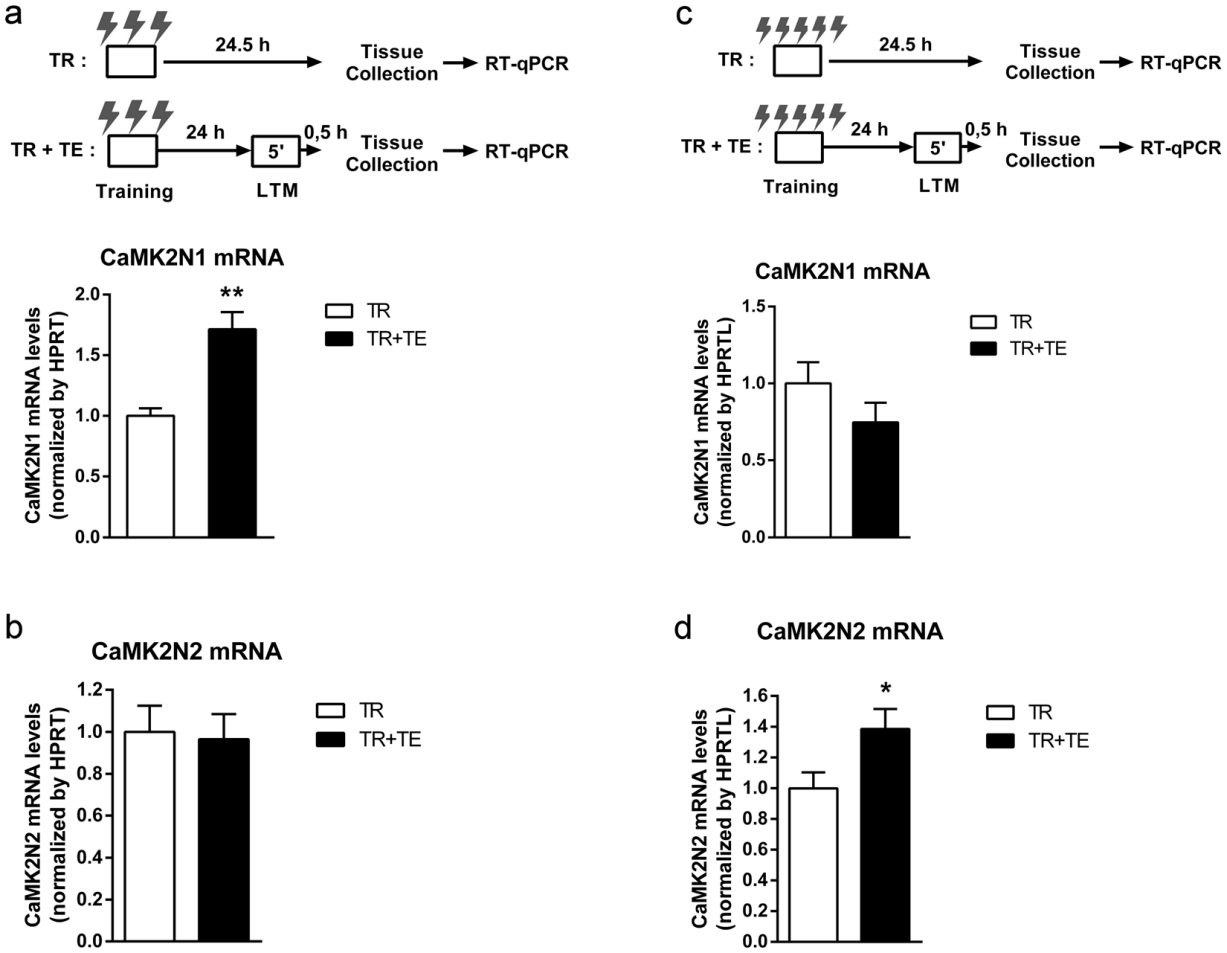
Effect of contextual fear memory retrieval on CaMK2N1 and CaMK2N2 dorsal hippocampi mRNA levels. (**a**) Animals (n = 10) were conditioned with 3 foot shocks (0.7 mA, 2 s). Half of the conditioned animals retrieved contextual fear LTM for 5 minutes 24 hours after conditioning (TR + TE). All animals were sacrificed 24.5 h after conditioning and mRNA expression in dorsal hippocampi was studied using RT-qPCR analysis. Retrieval of contextual fear LTM significantly upregulated CaMK2N1 expression. (**b**) CaMK2N2 mRNA levels were not altered by reactivation of contextual fear LTM. (**c**) A different cohort of animals (n = 12) was conditioned with 5 foot shocks (0.7 mA, 2 s) and, as in panel (**a**), half of the animals retrieved contextual fear LTM. CaMK2N1 expression did not change by retireval of this stronger contextual fear LTM. (**d**) However, CaMK2N2 mRNA levels were significantly increased by LTM retrieval. Hence, the intensity of the unconditioned stimulus influences the effect that memory retrieval has on the expression of the two endogenous CaMKII inhibitors. Mean and S.E.M.; *P < 0.05; **P < 0.01.

Previously, we showed that CFC transiently increases the expression levels of CaMK2N1 and CaMK2N2^11, 12^. However, the impact of contextual fear LTM retrieval on the levels of CaMK2N1 and CaMK2N2 mRNA was unknown. Thus, we studied whether hippocampal CaMK2N1 and CaMK2N2 mRNA expression differed in mice with retrieval of contextual fear LTM versus mice without retrieval (Fig. 2a). It is important to note that different from our previous publications^11, 12^ these experiments were designed to look for retrieval-induced changes in CaMK2N1 and CaMK2N2 mRNA levels. Using three foot shocks, the comparison revealed a significant increase in CaMK2N1 mRNA levels (F_1,9_ = 21.8; P < 0.01), by about 70% in the group subjected to LTM retrieval (Fig. 2a). This increase is of comparable magnitude to the ~50% increase in CaMK2N1 mRNA levels observed after CFC training, reported in previous publication by our group^11^. A retrieval-induced upregulation was not found for CaMK2N2 in these conditions (F_1,9_ = 0.04; P = 0.84) (Fig. 2b). On the other hand, conditioning mice with more shocks resulted in a retrieval-induced increase in CaMK2N2 mRNA levels (F_1,11_ = 5.4; P = 0.04) (Fig. 2d) with no effect on CaMK2N1 mRNA (F_1,11_ = 1.8; P = 0.20) (Fig. 2c). Therefore, CaMK2N1 may play a role in the maintenance of a weaker LTM after retrieval, while CaMK2N2 might be required for maintaining stronger LTM after retrieval.

### CaMK2N1 knockdown leads to impairment of contextual fear LTM after retrieval

**Figure 3:**
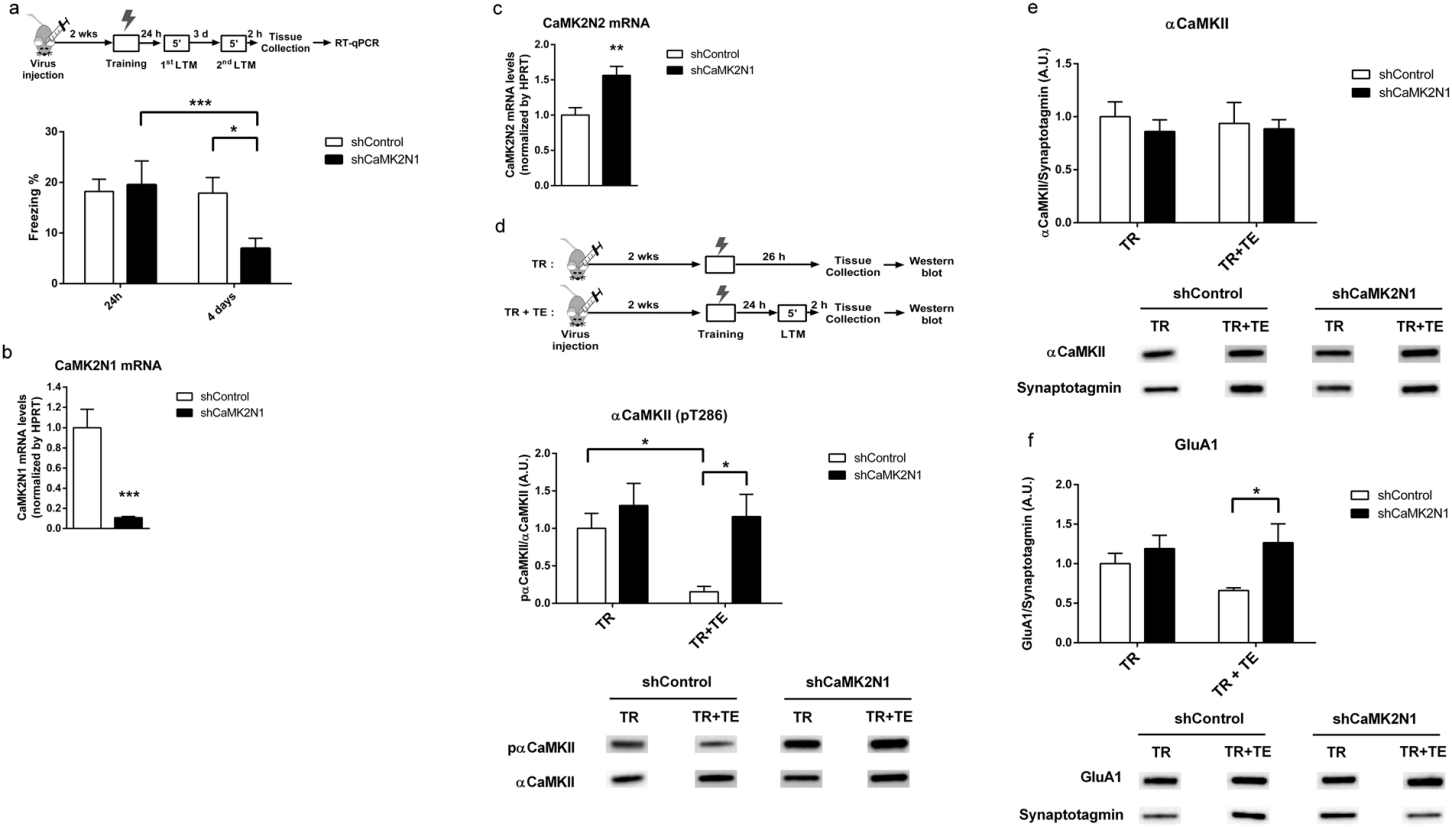
CaMK2N1 expression is necessary for contextual fear LTM maintenance after retrieval and regulates synaptic CaMKII T286 autophosphorylation and GluA1 levels. (**a**) Dorsal hippocampi of mice were transfected with rAAV expressing either control shRNA (n = 15) or CaMK2N1 shRNA (n = 11). Contextual fear LTM was determined one day after conditioning and for a second time 4 days after conditioning. CaMK2N1 shRNA expression in hippocampus impaired contextual fear LTM after retrieval. (**b**) All mice in (**a**) had a significant knockdown of CaMK2N1 mRNA expression in hippocampus. (**c**) In this cohort of mice, knockdown of CaMK2N1 caused a significant increase in CaMK2N2 mRNA expression. (**d**) To understand the retrieval-induced effects of CaMK2N1 knockdown mice were treated with shCaMK2N1 rAAV treatment and trained and tested (TR + TE) in the CFC paradigm or just trained (TR). Dorsal hippocampi samples were collected 2 hours after retrieval of contextual fear LTM, and protein samples prepared for western blots. Retrieval of contextual fear LTM significantly decreased αCaMKII autophosphorylation at T286 in synaptosomes (shControl: TR, n = 7, TR + TE, n = 5). This decrease in T286 autophosphorylation was impaired by shCaMK2N1 expression (TR, n = 7; TR + TE, n = 7). (**e**) shCaMK2N1 expression did not impact on αCaMKII protein levels (shControl: TR, n = 7, TR + TE, n = 6; shCaMK2N1: TR, n = 7; TR + TE, n = 7) (see statistics on Supplementary Table S4). (**f**) Quantification of GluA1 synaptosomal levels of the same cohort of animals revealed a retrieval-dependent increase due to CaMK2N1 knockdown (shControl: TR, n = 6, TR + TE, n = 5; shCaMK2N1: TR, n = 7; TR + TE, n = 7). Example immunoblotting staining for these proteins can be found below each graph and in Supplementary Fig. S7. Mean and S.E.M.; *P < 0.05; **P < 0.01; ***P < 0.001.

Since CaMK2N1 expression is upregulated after retrieval of relatively weak LTM, we tested whether knockdown of CaMK2N1 expression impairs contextual fear LTM after retrieval of one-trial contextual fear LTM (Fig. 3a). Considering that the experiments presented in Fig. 2 revealed that a low number of shocks favors retrieval-induced expression of CaMK2N1, and our aim was to investigate the function of this specific CaMKII endogenous inhibitor, we used only one foot-shock in the experiments shown in Fig. 3. After transfection of dorsal hippocampi with control or CaMK2N1 shRNA rAAV, mice were conditioned and tested for contextual fear LTM after one day followed by a second LTM test 4 days after conditioning. Two-way ANOVA with repeated measures showed a significant effect of memory tests (F_1,24_ = 9.0; P < 0.01) and a significant interaction between the memory tests and rAVV treatment (F_1,24_ = 8.1; P < 0.01). Student–Newman–Keuls (SNK) *post-hoc* comparisons revealed that CaMK2N1 shRNA expression significantly impaired contextual fear LTM only in the second LTM test in comparison with the control group (q = 3.4; P < 0.05). Further, there was a significant difference between first and second LTM test only within the mice expressing CaMK2N1 shRNA (q = 5.4; P < 0.001) (Fig. 3a). The same behavioral phenotype was observed in an independent replica experiment (Supplementary Fig. S3). Finally, it was confirmed that CaMK2N1 mRNA expression was substantially reduced in the behaviorally tested mice (F_1,25_ = 17.7; P < 0.001) (Fig. 3b). We found that in CaMK2N1 shRNA expressing mice CaMK2N2 mRNA expression was significantly elevated (F_1,25_ = 13.3; P < 0.01) (Fig. 3c). A correlation analysis suggests that the increased CaMK2N2 expression might be a compensation for the down-regulation of CaMK2N1 expression in these mice (Supplementary Fig. S4). That being the case, the compensation was not enough to avoid the retrieval-induced memory impairment after CaMK2N1 knockdown. Effects of this CaMK2N2 increase will be addressed later in this article.

### CaMK2N1 knockdown affects synaptic αCaMKII T286 autophosphorylation and GluA1 levels, after LTM retrieval

To study the impact of CaMK2N1 knockdown on CaMKII function we analyzed αCaMKII autophosphorylation at T286 in synaptosomes from dorsal hippocampi. T286 autophosphorylation levels were compared between mice that underwent CFC (TR group) and mice that underwent CFC and LTM retrieval (TR + TE group) (Fig. 3d). Two-way ANOVA analysis of T286 autophosphorylation levels showed a significant effect of shCaMK2N1 expression (F_1,26_ = 6.6; P < 0.05) (Fig. 3d). *Post-hoc* comparisons revealed a significant decrease in T286 autophosphorylation between TR + TE and TR mice, only for the control treatment (q = 3.1; P < 0.05), and not for the shCaMK2N1 treatment (q = 0.6; P = 0.67). A significant difference in T286 autophosphorylation levels was also observed between control and shCaMK2N1 treatment only for TR + TE mice (q = 3.7; P < 0.05), but not within TR animals (q = 1.2; P = 0.382). Thus, LTM retrieval reduced T286 autophosphorylation levels in control mice, but not in CaMK2N1 knockdown mice. This retrieval-induce change in T286 autophosphorylation may occur at synapses only, as it was not detected in the cytoplasmic fraction (Supplementary Fig. S5). Total αCaMKII protein levels were not affected by CaMK2N1 knockdown (Fig. 3e and Supplementary Fig. S6).

To look for downstream effects of CaMK2N1 knockdown and the observed increase in T286 autophosphorylation, GluA1 levels were quantified in the same synaptosomal samples (Fig. 3f). Two-way ANOVA revealed a significant effect of CaMK2N1 knockdown in GluA1 levels (F_1,25_ = 4.9; P = 0.03). *Post-hoc* comparisons showed a significant increase in GluA1 levels of shCaMK2N1 animals compared with shControl only when animals underwent memory retrieval (q = 3.2; P = 0.03), but not when they were only trained (q = 1.0; P = 0.44). There was no significant difference between TR and TR + TE animals when comparing within shControl (q = 1.7; P = 0.220) or shCaMK2N1 (q = 0.4; P = 0.757) animals.

### Increased hippocampal CaMK2N2 expression impairs LTM formation but does not affect LTM maintenance after retrieval

**Figure 4:**
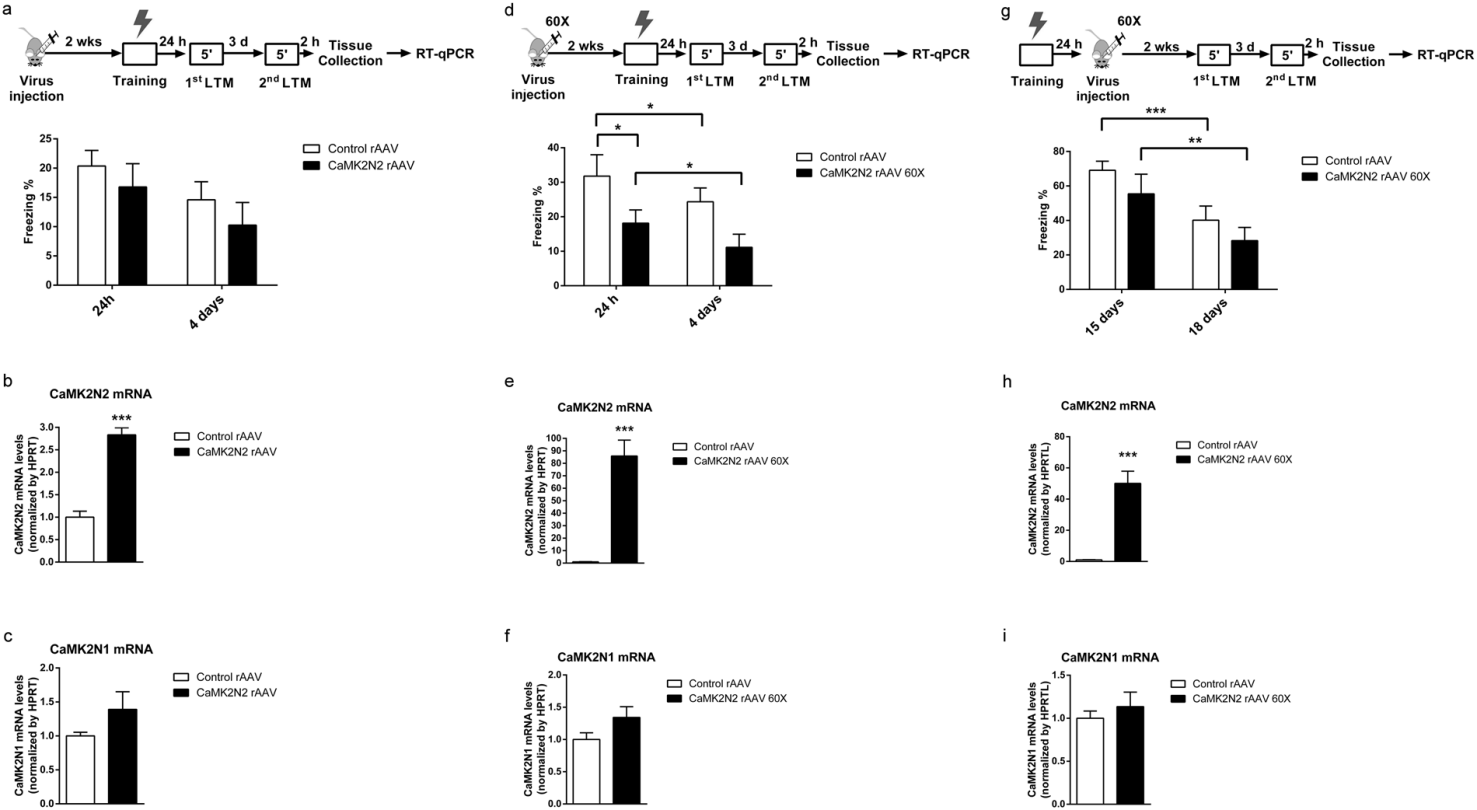
Overexpression of CaMK2N2 in dorsal hippocampi impairs memory formation but does not impair contextual fear LTM maintenance. (**a**) Dorsal hippocampi of mice were transfected with rAAV expressing either ZsGreen (n = 13) or CaMK2N2 (n = 11) under control of a synapsin 1 promoter. Contextual fear LTM was determined one day and 4 days after conditioning. No significant difference was observed. (**b**) Mice studied in (**a**) overexpressed CaMK2N2 mRNA in dorsal hippocampus (Control rAAV, n = 11; CaMK2N2 rAAV, n = 10). (**c**) Overexpression of CaMK2N2 mRNA did not alter CaMK2N1 mRNA levels (Control rAAV, n = 9; CaMK2N2 rAAV, n = 9). (**d**) Another cohort of animals was subjected to the same experimental design as in (**a**) with the exception that these animals were treated with a 60x more concentrated version of the CaMK2N2 rAAV solution (1.07 × 10^13^ GC/ml). In these mice CaMK2N2 overexpression reduced freezing scores in the first memory test, indicating impaired memory formation (Control rAAV, n = 11; CaMK2N2 rAAV, n = 10). (**e**) RT-qPCR analysis confirms effective overexpression of CaMK2N2 in dorsal hippocampi of CaMK2N2 rAAV 60x animals (n = 10) compared to Control rAAV (n = 11). (**f**) CaMK2N2 rAAV 60x treatment did not affect CaMK2N1 mRNA levels (Control rAAV, n = 11; CaMK2N2 rAAV, n = 10). (**g**) A final cohort of animals was first conditioned in the CFC paradigm and 24 hours later treated with the 60x CaMK2N2 rAAV solution. A period of 2 weeks was waited to allow the genetic information from the vector to be expressed, after which the fear memory of the context was tested twice. The two tests were apart from each other by 3 days, therefore they were executed 15 days and 18 days after CFC conditioning. No significant effect of 60x CaMK2N2 rAAV treatment was observed in the freezing behavior (Control rAAV, n = 8; CaMK2N2 rAAV, n = 7). (**h**) CaMK2N2 mRNA levels were increased after 60x CaMK2N2 rAAV treatment (Control rAAV, n = 8; CaMK2N2 rAAV, n = 7). (**i**) CaMK2N2 overexpression did not affect CaMK2N1 mRNA levels (Control rAAV, n = 8; CaMK2N2 rAAV, n = 7). Mean and S.E.M.; *P < 0.05; **P < 0.01; ***P < 0.001.

Considering that the animals that presented a retrieval-induced LTM impairment after CaMK2N1 knockdown (Fig. 3a) also had an increase in CaMK2N2 expression (Fig. 3c), the LTM impairment observed could have been due to the increased CaMK2N2 expression. To study this possibility, we prepared a rAAV that overexpresses CaMK2N2 under control of the synapsin 1 promoter (see Supplementary Methods). After infection of dorsal hippocampi with control or CaMK2N2-expressing rAAVs, mice were conditioned and tested for contextual fear LTM after one day followed by a second LTM test 4 days after conditioning (Fig. 4a). Analysis of the freezing scores using two-way ANOVA with repeated measures indicated that transfection with the CaMK2N2-expressing rAAV did not lead to an effect of treatment (F_1,22_ = 3.0; P = 0.09) and there was no significant interaction between treatment and the memory tests (F_1,22_ = 0.3; P = 0.59). mRNA expression analysis confirmed that the CaMK2N2-expressing rAAV significantly elevated CaMK2N2 expression by about ~2.5 fold (F_1,20_ = 81.1; P < 0.001) (Fig. 4b), which is comparable to the ~1.5 fold increase observed as a compensation after CaMK2N1 knockdown treatment (Fig. 3c). CaMK2N2 overexpression treatment did not alter CaMK2N1 expression (F_1,19_ = 3.2; P = 0.09) (Fig. 4c). Hence, the reduced CaMK2N1 expression directly caused the contextual LTM impairment after retrieval, not the compensatory increase in CaMK2N2 expression (Fig. 3a).

Using a 60x more concentrate version of the CaMK2N2-expressing rAAVs solution we obtained an ~80-fold increase in CaMK2N2 mRNA levels in dorsal hippocampus (Fig. 4e). We conditioned this cohort of animals and tested their LTM 24 hours and 4 days after conditioning (Fig. 4d). A two-way ANOVA with repeated measures revealed a significant effect of CaMK2N2 overexpression (F_1,19_ = 4.7; P = 0.04) and a significant effect of the memory tests (F_1,19_ = 10.3; P = 0.004), but no interaction between these factors (F_1,19_ = 0.01; P = 0.92). SNK *post-hoc* comparisons revealed a significant decrease of freezing behavior in CaMK2N2 overexpression animals, when compared with control group, in the 24-hour memory test (q = 2.9; P = 0.04), and a trend in the 4-day memory test (q = 2.8; P = 0.05). This result suggests that a high-level CaMK2N2 overexpression in the hippocampus impairs LTM formation. SNK planned comparisons also revealed a significant effect of the LTM tests within the control rAAV (q = 3.4; P = 0.02) and within the CaMK2N2 rAAV 60x group (q = 3.0; P = 0.04). Two hours after the last LTM test hippocampal samples were collected for RT-qPCR analysis. A significant increase in CaMK2N2 mRNA levels (F_1,20_ = 48.6; P < 0.001) (Fig. 4e) were observed with no significant effect on CaMK2N1 mRNA expression (F_1,20_ = 3.1; P = 0.09) (Fig. 4f), thereby confirming the specificity and efficiency of the rAAV treatment.

To test for any effect of CaMK2N2 overexpression on contextual fear LTM maintenance the rAAV was injected 24 hours after CFC and contextual fear LTM was tested 14 and 17 days after the infection (Fig. 4g). No effect of rAAV treatment on LTM was observed (F_1,13_ = 1.4; P = 0.25) under these conditions. There was a significant effect of the repeated LTM tests (F_1,13_ = 39.1; P < 0.001), but this effect presented no interaction with the rAAV treatment (F_1,13_ = 0.04; P = 0.84). SNK planned comparisons also showed no significant effect of high- level CaMK2N2 overexpression in the first (q = 1.6; P = 0.25) and second (q = 1.4; P = 0.32) LTM test. There was only a significant difference between the freezing scores on the first and second LTM test within the control rAAV group (q = 6.6; P < 0.001) and within the CaMK2N2 rAAV 60x group (q = 5.8; P = 0.001). Therefore, CaMK2N2 overexpression after training had no effect on LTM maintenance. RT-qPCR analysis confirmed substantial CaMK2N2 overexpression (F_1,14_ = 45.9; P < 0.001) (Fig. 4h) with no effect on CaMK2N1 expression (F_1,14_ = 0.5; P = 0.46) (Fig. 4i).

## Discussion

Investigating the function of the endogenous CaMKII inhibitors in learning and memory, we found that CaMK2N1 is essential for LTM maintenance after retrieval. Thus, our finding suggests that prevention of LTM impairment after retrieval requires sophisticated fine-tuning of CaMKII activity. Consistently, previous transgenic mouse studies have shown that elevated CaMKII activity at the time of retrieval leads to LTM loss^13^. We observed a reduction of T286 autophosphorylation of αCaMKII within dorsal hippocampus after LTM retrieval and this process required CaMK2N1. The CaMK2N1-dependent regulation of T286 autophosphorylation was not predicted by earlier *in vitro* work with the inhibitory domain of CaMK2N1, CN21, and purified αCaMKII^14^, which reported that T286 autophosphorylation would only be “mildly affected” by CaMK2N1. This discrepancy with our findings is likely to be a result of the difference between studying the whole CaMK2N1 protein instead of a 21 amino acid fragment and/or *in vivo* versus *in vitro* studies. Since CaMK2N1 does not physically block the T286 site, as it binds to αCaMKII in a different area^14^, it is likely that CaMK2N1 regulates T286 phosphorylation by regulating αCaMKII kinase activity.

We also found that CaMK2N1 knockdown results in a retrieval-induced increase in synaptic GluA1 levels. It is known that T286 autophosphorylation augments αCaMKII activity^15, 16^ and that the kinase phosphorylates the scaffold protein stargazin, trapping GluA1-containing AMPA receptors within the synapse^6^. Therefore, the impairment in retrieval-induced decrease of T286 autophosphorylation due to CaMK2N1 knockdown, could explain a retrieval-induced increase in synaptic GluA1 levels. Consistently, GluA1 levels in the synapse have been shown before to be decreased in the dorsal hippocampus of mice when contextual fear memory is retrieved 24 hours after conditioning^17^. In contrast, memory extinction increases GluA1 levels in different regions of the brain including the dorsal hippocampus^18, 19^. Extinction also requires the T286 autophosphorylation^3^. Additionally, forgetting of spatial working memory is associated with increased T286 autophosphorylation and GluA1 levels^20^. Hence, it is possible that the retrieval-induced increase in T286 autophosphorylation and GluA1 levels due to CaMK2N1 knockdown disrupted the normal chain of molecular events induced by memory retrieval, facilitating extinction or memory loss. Nonetheless, more studies are necessary to link directly the observed effects of CaMK2N1 knockdown on GluA1 levels to the effects on LTM maintenance.

Another possible explanation for the memory phenotype observed after CaMK2N1 knockdown is an impairment in memory destabilization/reconsolidation process. Memory destabilization, part of the memory reconsolidation hypothesis, has been shown to be necessary for memory maintenance after retrieval^20^. It is possible that the retrieval-induced decrease in αCaMKII T286 autophosphorylation and synaptosomal GluA1 levels observed in control animals is part of the destabilization of a memory. In this case, the retrieval-induced increase T286 autophosphorylation and GluaA1 levels observed after CaMK2N1 knockdown might result in memory impairment due to blocking of memory destabilization. It is also possible that CaMK2N1 knockdown results in impairment of other steps of memory reconsolidation, however, more experiments are necessary to test this idea. Nevertheless, the clear separation of any retrieval-induced amnesic phenotype between a reconsolidation impairment and an increased memory extinction is still a matter of debate (see Trent, *et al*.^21^ and Almeida-Correa and Amaral^22^ for a more complete discussion on the subject).

Additionally, other molecular mechanisms could also be involved with the retrieval-dependent LTM impairment due to CaMK2N1 knockdown. For example, CaMKII activity regulates protein degradation at synapses and LTM destabilization following retrieval^21^ and CaMKII interaction with F-actin has also been proposed to play an important structural role in LTM^23^. Enhanced adult neurogenesis in dentate gyrus has also been linked with forgetting^23^, but the short time course of our experiments makes it unlikely that CaMK2N1 regulates it.

Taken together, we establish for the first time a specific role for CaMK2N1 in learning and memory. CaMK2N1 in dorsal hippocampus prevents fear LTM loss after retrieval by blocking T286 autophosphorylation of αCaMKII. It is possible that CaMK2N2 has a related function, since its expression is regulated after retrieval of a strong rather than weak LTM. Follow-up studies are needed to establish how CaMK2N1 and CaMK2N2 memory functions relate to each other. Furthermore, more studies are necessary to address the effects of the retrieval-induced changes on CaMK2N1 and CaMK2N2 expression on βCaMKII function. It is also important to consider that the endogenous inhibitors of CaMKII have been show to also block CaMKII autophosphorylation at the T305 site^13^. A retrieval-dependent function for such CaMKII phosphorylation site has never been shown, nevertheless more experiments are important to approach this question. CaMK2N1 is also known to inhibit CaMKII binding to Cav2.1 calcium channels^24^. The relevance of such inhibition for memory maintenance is still unknown.

Treatment of post-traumatic stress disorder and drug addiction by facilitating memory extinction and/or impairing memory reconsolidation have been suggested and are a currently a major field of investigation^25, 26^. CaMK2N1 could be a possible molecular target for treating these diseases.

A relatively small increase of CaMK2N2 mRNA levels (~2.5 fold increase) in dorsal hippocampus did not affect contextual fear LTM. This result of CaMK2N2 overexpression confirms the independence of retrieval-induced LTM impairment from the CaMK2N2 mRNA increase observed concomitantly with CaMK2N1 knockdown. Higher levels of CaMK2N2 overexpression (50–80 fold increase) impaired LTM formation, but did not affect well-established contextual fear LTM. These results are in accordance with Buard, *et al*.^4^ reporting that CaMKII inhibition by tatCN21 administration impairs contextual fear memory formation but not LTM maintenance. Overall, the data presented here corroborate with the hypothesis that endogenous inhibition of CaMKII is necessary for LTM maintenance after retrieval. Thus, CaMKII inhibition by CaMK2N2 overexpression or tatCN21 treatment^4^ are not effective ways of studying CaMKII’s roles after LTM retrieval as they only increase the necessary inhibition of CaMKII.

Mutation R495X in the FUS gene (also known as FUS-ΔNLS), an amyotrophic lateral sclerosis (ALS)/frontotemporal dementia (FTD) causing mutation^27^, has been shown, *in vitro*, to induce overexpression of CaMK2N2^28^. We have observed that hippocampal CaMK2N2 overexpression impairs memory formation. ALS/FTD patients present cognitive impairments, with lower scores in tests of working memory, executive function, face recognition, and others^29–31^. It is possible that CaMK2N2 overexpression might be involved in the cognitive symptoms of ALS/FTD. Additionally, some forms of ALS/FTD includes the formation of inclusions with TAR DNA binding protein-43 (TDP-43) and RNA molecules in neurons^32, 33^. Wang, *et al*.^34^ has reported that mRNAs for αCaMKII are among the mRNAs trapped in TDP-43 inclusions. Also, loss of function in progranulin protein, an animal model that induces the appearance of TDP-43 inclusions, has been show to increase T286 autophosphorylation of αCaMKII^35^. Based on these evidences, more studies on the roles of CaMKII and CaMK2N2 in ALS’s progression and cognitive symptoms are important.

## Methods

### Animals

Male adult (10–12 week-old) C57BL/6J mice were group-housed in a 12-hour light-dark cycle with food and water ad libitum. For the experiments animals were divided between the treatments so that each cage would include animals from all the treatments. During the collection of behavioral and molecular data the experimenter was blinded to the animal’s treatment. Experiments were repeated once or twice. All experiments were undertaken in accordance with local ethical reviewing at King’s College London, UK and in agreement with the UK Animals (Scientific Procedures) Act 1986.

### Viral vectors

Three plasmids were generated in our laboratory, been those used for knocking down CaMK2N1, overexpressing CaMK2N2 or a control plasmid. Those plasmids were inserted in rAAV, serotype 2/9 (Penn VectorCore, University of Pennsylvenia, USA), which were used as vectors for gene expression manipulation in the dorsal hippocampus of mice. More details about the plasmids can be found in Supplementary Methods.

### Stereotaxic surgery

Mice were subjected to stereotaxic surgery for bilateral injection of the rAAVs into the dorsal hippocampus. The coordinates for injection were anterior-posterior: +2.1 mm; medio-lateral: ±1.5 mm; dorso-ventral: −1.8 mm from bregma. The total volume of injection was 0.75 μl of viral vector solution at a rate of 0.25 μl/min. More details on the surgery can be found on Supplementary Methods.

### Contextual fear conditioning

Prior to behavioral protocol each animal was handled for 1 minute for 5 days. For CFC the mouse was placed into the conditioning chamber (30.5 cm × 24.1 cm × 21.0 cm) (MedAssociates, VFC-008-LP), after 40 s one, three or five mild foot shocks (0.7 mA for 2 s) were given and after further 30 s the mouse was returned to its home cage (see, ref. 35). To test for contextual fear memory, the mouse was returned to the same conditioning chamber and freezing behavior was scored for 2 s in 5 s intervals over a 5 min period. Freezing was positively scored if no movement other than respiratory movements was observed. To control the shock and record animals’ behavior VFreeze software was used (MedAssociates). Scoring was done blind to treatment. In the first two experiments scoring was done twice by two different experimenters to test the precision of scoring.

### RT-qPCR

In order to assess CaMK2N1 and CaMK2N2 mRNA expression levels RT-qPCR was used. RT-qPCR was executed following the procedures described in Lepicard, *et al*.^11^. Triplicates were used for each sample. A detailed description of the protocol and the primers used can be found in Supplementary Methods.

### Western blot

Western blot technique was performed in order to quantify the levels of αCaMKII, GluA1 and αCaMKII T286 autophosphorylation. Crude synaptosome fractions were obtained following protocols previously published^36–38^. Quantification of bands was obtained after exposure in the linear range. Example images can be seen on Supplementary Fig. S7. A full description of the protocol used can be seen on Supplementary Methods.

### Fluorescence microscopy

The rAAVs used had either ZsGreen or GFP as reporter genes. Expression of these fluorescent proteins after injection was assessed as a control for the surgery precision and viral transfection (Supplementary Fig. S1). A detailed explanation of the fluorescence microscopy procedure used is presented at Supplementary Methods.

### Statistical analysis

Data were checked for normal distribution by Q-Q plot analysis, Kolmogorov-Smirnov and/or Shapiro-Wilk normality test. In case of non-normal distribution outliers were excluded from experiment as a normalization strategy. Outliers were defined as values that fall more than 1.5 times the interquartile range above the third quartile or below the first quartile. Animals treated with shCaMK2N1 rAAV were excluded from the experiment if the RT-qPCR analysis showed that the level of CaMK2N1 expression was an outlier in the group. This was considered to be an indication of treatment failure. The same principle was applied for animals treated with CaMK2N2-expressing rAAV but with RT-qPCR analysis of CaMK2N2 levels of expression. Animals which did not freeze in the memory test were excluded for the western blot analysis, since the aim was to look for molecular alterations related to memory changes. One-way ANOVA was used for analysis of dependent variables if the experiment had only one independent variable. In experiments with two independent variables, dependent variables were analyzed by two-way ANOVA, with repeated measures if necessary (for example when comparing behavioral results from first and second memory test). SNK test was used for *post-hoc* comparison as probing method for determining significant differences between different groups, once ANOVA revealed a significant difference.

## Electronic supplementary material

Supplementary information
