# Supplementary material for: Prevention of long-term memory loss after retrieval by an endogenous CaMKII inhibitor
Source: Sci Rep. 2017 Jun 22;7:4040. doi: 10.1038/s41598-017-04355-8 (PMC5481336; doi:10.1038/s41598-017-04355-8)
Supplement: Supplementary file 1 — Supplementary information [file 41598_2017_4355_MOESM1_ESM.pdf]

Prevention of long-term memory loss after retrieval by an endogenous CaMKII inhibitor

Fabio Antonio Vigil, Keiko Mizuno, Walter Lucchesi, Victoria Valls Comamala, Karl Peter Giese

Supplementary Methods

1. Viral vectors

In order to knock-down CaMK2N1 the viral vector used expressed shRNA under control of the U6 promoter Supplementary Table S1). This CaMK2N1 knockdown vector also expressed ZsGreen under control of the synapsin 1 promoter (a kind gift of Dr. Marco Peters, Dart Neuroscience, San Diego, USA). Virus titre was 1.39×10^{13} genome copies (GC)/ml. The control vector expressed a scrambled shRNA sequence under control of the U6 promoter and it also expressed ZsGreen under control of the synapsin 1 promoter. The titre was 1.2×10^{13} GC/ml. To manipulate the expression of CaMK2N2 we have used a rAAV bicistronic vector based on the synapsin 1 promoter driving the independent expression of the human CaMK2N2 gene and the GFP reporter gene, both separated by an internal ribosome entry site (IRES) sequence. The titre was 1.79×10^{11} GC/ml. The sequences used in all viral vectors are shown on Supplementary Table S1.

2. Stereotaxic surgery

The stereotaxic surgery was performed using a stereotaxic frame David Kopf Instrument, Tujunga, CA; Model 963 Ultra Precise Small Animal Stereotaxic Instrument. For viral vector injection a 2.5 µl Hamilton syringe (Hamilton Medical, Reno, NV; 87942) and a bilateral microsyringe pump controller (UltraMicroPump (UMP3) with SYS-Micro4 Controller - World Precision Instruments, Sarasota, UK; UMP3-1) were used. After recovery from anaesthesia the mouse was returned to its home cage. Mice were left two weeks without been subjected to any procedure.

3. Fluorescence microscopy

For fluorescence microscopy the mouse was perfused with 4 % w/v paraformaldehyde (PFA) (Sigma-Aldrich, St. Louis, MO, USA; P6148) in PBS. After dissection and storage overnight the brain was incubated in 30 % w/v sucrose (Calbiochem® - Merck Millipore, Darmstadt, Germany; 5737) solution diluted in PBS (Life Technologies, Paisley, UK; 10010-023) with 0.05 % sodium azide (Sigma-Aldrich,

St. Louis, MO, USA; 438456) at 4°C. Coronal 40 µm sections were mounted in Superfrost® plus slides (VWR International, Radnor, PA, USA; 48311-703), and covered with Vectashield® mounting medium for fluorescence with 4',6-diamidino-2-phenylindole (DAPI) (Vector Laboratories, Inc., Burlingame, CA, USA; H-1200) and cover glass (22 mm x 64 mm) (VWR International, Radnor, PA, USA; 631-0880). The sections were analysed with a Zeiss AxioImager.Z1 fluorescent microscope (Zeiss, Oberkochen, Germany) with AxioVision software (Zeiss, Oberkochen, Germany) and a monochrome AxioCam MRm Rev.3 camera (Zeiss, Oberkochen, Germany). Images from GFP or ZsGrenn fluorescence were merged with DAPI florescence images, from the same area, by image analyses software ImageJ (National Institute of Mental Health, Bethesda, MD, USA). Examples are shown in Figure S1.

4. mRNA expression analysis

In order to quantify mRNA expression levels hippocampi were dissected and for each hemisphere divided into dorsal and ventral. The sample was homogenized in 600 µl of TRIzol® buffer (Life Technologies, Paisley, UK; 15596-026) using polytron homogenizer PowerGen 125 (Fisher Scientific International, Inc., Hampton, NH, USA; 12396727) 30 to 45 seconds. After chloroform extraction the total RNA was precipitated. The quality of the RNA samples was assessed with the BioSpectrum® imaging system (UVP, Upland, CA, USA) after electrophoresis. cDNAs were synthesised with SuperScript® II Reverse Transcriptase (Life Technologies, Paisley, UK; 18064-014), oligo(dT)12-18 primer (Life Technologies, Paisley, UK; 18418-012), deoxynucleotide (dNTP) mix (Qiagen, Hilden, Germany; 201900), dithiothreitol (DTT) (Life Technologies, Paisley, UK; D-1532), RNasin® Inhibitor (Promega, Fitchburg, WI, USA; N2115) and first strand buffer (Life Technologies, Paisley, UK; 18064-014) according to published protocol[7]. For amplification of CaMK2N1, CaMK2N2 and the housekeeping gene hypoxanthine phosphoribosyl transferase (HPRT) specific primers were previously designed using Primer Express 2.0 software (Applied Biosystems, Foster City, CA, USA) and tested for specificity by sequencing of the amplification product. The primer sequences are shown in Supplementary Table S2. Dilution of the cDNA samples was determined by equal amplification efficiencies of both the template and HPRT throughout a range of cDNA dilutions. Optimum sample dilution was defined as a middle point in the line which the equation describing it had an “a” value smaller then 0.1. RT-qPCR was performed in 0.2 ml non-skirted low profile 96-well PCR plate (Life Technologies, Paisley, UK; AB-0700) capped with qPCR caps (Life Technologies, Paisley, UK; AB-

0866). Each well contained 5 µl of the cDNA template solution and 15 µl of RT-qPCR solution, composed of forward and reverse primers, nuclease free water and the fluorescent marker 2x qPCR MasterMix with SYBRGreen and ROX (PrimerDesign Ltd., Hants, UK; Precision-R). The amplification of the PCR product was continuously measured by Chromo4™, real-time PCR detector using a DNA Engine® (Bio-Rad, Hemel Hempstead, UK; CFB-3240) during 45 cycles. The reaction started with one step of 95°C for 10 minutes, followed by 45 cycles of 20 seconds at 95°C, 20 seconds at 60°C and 20 seconds at 72°C. The number of cycles needed to reach exhaustion of the reaction, as well as, the melting points of paired cDNA strands made in the reactions was calculated for each well with the analysis software Opticon Monitor, version 3.1.32 (Bio-Rad, Hemel Hempstead, UK). Every sample was amplified in triplicates, and mean threshold cycle (CT) was determined. The ΔC_t method was used to compare mRNA expression levels[7].

5. Western blot analysis

For protein extraction the right and left hippocampus of each mouse were combined into one sample. Samples were homogenized in homogenization buffer containing 0.32 M sucrose (Calbiochem® - Merck Millipore, Darmstadt, Germany; 5737), 1 mM $NaHCO_3$ (Sigma-Aldrich, St. Louis, MO, USA; S6014), 1 mM $MgCl_2$ (Sigma-Aldrich, St. Louis, MO, USA; M2670), 10 mM Hepes (Sigma-Aldrich, St. Louis, MO, USA; H3375), 1.5% v/v protease inhibitor cocktail (Sigma-Aldrich, St. Louis, MO, USA; S8820), 1% v/v phosphatase inhibitor cocktail 2 (Sigma-Aldrich, St. Louis, MO, USA; P5726) and 1% v/v phosphatase inhibitor cocktail 3 (-Sigma-Aldrich, St. Louis, MO, USA; P0044). Homogenization was made on ice and by hand-douncing 20 times in a borosilicate glass tissue grinder (Kimble Chase, Vineland, NJ, USA; K885300/0002). After centrifugation at 4°C for 5 min at 2000 rpm the pellet was re-suspended in homogenization buffer; this fraction was P1. The supernatant fraction was collected and centrifuged at 13.000 rpm for 15 min at 4°C. The resulting supernatant was collected (cytosol fraction S2) and the pellet was re-suspended in homogenization buffer (crude synaptosomal fraction P2)[38-40]. Protein concentration of each sample was determined with the Pierce™ BCA Protein Assay Kit (Life Technologies, Paisley, UK; 23225). Samples (20 µg) were run on 18-well Criterion™ TGX™ AnykD™ gels (Bio-Rad Laboratories, Hemel Hempstead, UK; 5671124). For protein transfer nitrocellulose membrane from Immun-Blot® PVDF Membrane (Bio-Rad Laboratories, Hemel Hempstead, UK; 162-0177) was used. Membranes were blocked with 5 % w/v skim milk powder

(Merck Millipore, Darmstadt, Germany; 115363) diluted in tris-buffered saline and 0.05 % v/v Tween™ 20 (Fisher Scientific International, Inc., Hampton, NH, USA; BP337100) (TBST) solution. TBST solution was also used for washing the membranes. A detailed list of the primary antibodies used and secondary antibody dilution can be found in Supplementary Table S3. For detection of all the secondary antibodies the Pierce™ ECL western blotting substrate (Thermo Scientific, Waltham, MA, USA) was used. Quantification of bands was done after exposure in the linear range. Example images can be seen on Supplementary Fig. S7. For normalization the results of every protein of interest were divided by the results of a housekeeping protein from the same membrane or the total protein of interest, if the antibody of interest was targeting a phosphorylated protein.

Supplementary Table S1. shRNA sequences and CaMK2N2 sequence

Name	Sequence (5’ – 3’)	Targeted sequence
Scrambled shRNA	GGATCCTCGCTTGGGCGAGAGTAAGTTCAAGAGACTTA CTCTCGCCCAAGCGATTTTTTACTAGTGGATCC	No homology
ShCaMK2N1	GGATCCGAGCAAGCGCGTTGTTATTCTCAAGAGAAATA ACAACGCGCTTGCTCTTTTTTACTAGTGGATCC	See: https://www.ncbi.nlm.nih.gov/nuccore/NM_025451.2
CaMK2N2	GAATTCACCATGTCCGAGATCCTACCCTACGGTGAGGA CAAGATGGGCCGCTTCGGCGCAGACCCCGAAGGTTCC GACCTCTCTTTCAGCTGCCGCCTGCAGGACACCAACTC CTTCTTCGCTGGCAACCAGGCCAAGCGGCCCCCCAAG CTGGGCCAGATCGGCCGAGCCAAGAGAGTGGTGATCG AGGATGACCGGATAGACGACGTGCTGAAGGGGATGGG GGAGAAGCCTCCGTCCGGAGTGTAG	See: https://www.ncbi.nlm.nih.gov/gene/94032

Supplementary Table S2. RT-qPCR primer sequences

Gene	Direction	Sequence (5' – 3')	Concentration
HPRT	Forward	ATACAGGCCAGACTTTGTTGGATT	300nM
	Reverse	TCACTAATGACACAAACGTGATTCAA	300nM
CaMK2N1	Forward	GCTGAAAACCATGACCGACAA	300nM
	Reverse	AAGAATAAGAAAGGGAGTTGTGTAAC	300nM
CaMK2N2	Forward	CGCAGACCCCGAAGGTT	300nM
	Reverse	TTGCCAGCGAAGAAGGAGTT	900nM

Supplementary Table S3. Western blot antibodies

Protein	Manufacturer (product number)	Dilution	Secondary antibody dilution
αCaMKII	Chemicon (MAB8699)	1:100,000	1:30,000
pαCaMKII (T286)	Abcam (ab5683)	1:1000 / 1:10,000*	1:2000
Synaptotagmin	Sigma (S2177)	1:30,000	1:2000
Neuron specific enolase (NSE)	Millipore (AB951)	1:60,000	1:20,000
GluA1	Millipore (05-855R)	1:1000	1:2000

*Dilutions of 1:1000 and 1:10,000 were used for samples from S2 and P2 fractions, respectively.

Supplementary Table S4 Non-significant results

Non-significant results that were not presented in the main article can be found in this table.

Experiment	Data	Statistical Test	Groups	Result
CaMK2N1 knockdown with 2 memory tests (Figure 3a)	Freezing scores	Two-way ANOVA (effect of rAAV treatment)	shControl X shCaMK2N1	($F_{1,24}$=1.4; P=0.23)
		SNK comparisons	shControl X shCaMK2N1 (within 24 h)	(q=0.4 ; P=0.77)
			24 h X 4 days (within shControl)	(q=0.1 ; P=0.94)
CaMK2N1 knockdown in TR and TR+TE animals	αCaMKII levels in P2 fraction (Figure 3e)	Two-way ANOVA (effect of memory test)	TR X TR+TE	($F_{1,27}$=0.02; P=0.89)
		Two-way ANOVA (effect of rAAV treatment)	shControl X shCaMK2N1	($F_{1,27}$=0.4; P=0.49)
		Two-way ANOVA (interaction effect)	memory test X rAAV treatment	($F_{1,27}$=0.1; P=0.75)
	T286 phosphorylated αCaMKII levels in P2 fraction (Figure 3d)	Two-way ANOVA (effect of memory test)	TR X TR+TE	($F_{1,26}$=3.8; P=0.064)
		Two-way ANOVA (interaction effect)	memory test X rAAV treatment	($F_{1,26}$=1.8; P=0.185)
	GluA1 levels in P2 fraction (Figure 3f)	Two-way ANOVA (effect of memory test)	TR X TR+TE	($F_{1,25}$=0.5; P=0.468)
		Two-way ANOVA (interaction effect)	memory test X rAAV treatment	($F_{1,25}$=1.3; P=0.261)
CaMK2N2 overexpression with 1X rAAV solution (Figure 4a)	Freezing scores	Two-way ANOVA (effect of memory tests)	24 h X 4 days	($F_{1,22}$=11.2; P=0.003)*
		SNK comparisons	24 h X 4 days (within Control rAAV)	(q=2.9; P=0.051)
			24 h X 4 days	(q=1.5;

			(within CaMK2N2 rAAV)	P=0.29)
			Control rAAV X CaMK2N2 rAAV (within 24 h)	(q=0.5; P=0.71)
			Control rAAV X CaMK2N2 rAAV (within 4 days)	(q=0.9; P=0.52)

*There was a significant effect of the consecutive memory tests in this analysis. However, the effect was not related to the rAAV treatment and it was not confirmed by SNK comparisons (rows below), therefore it was ignored.

Supplementary Results

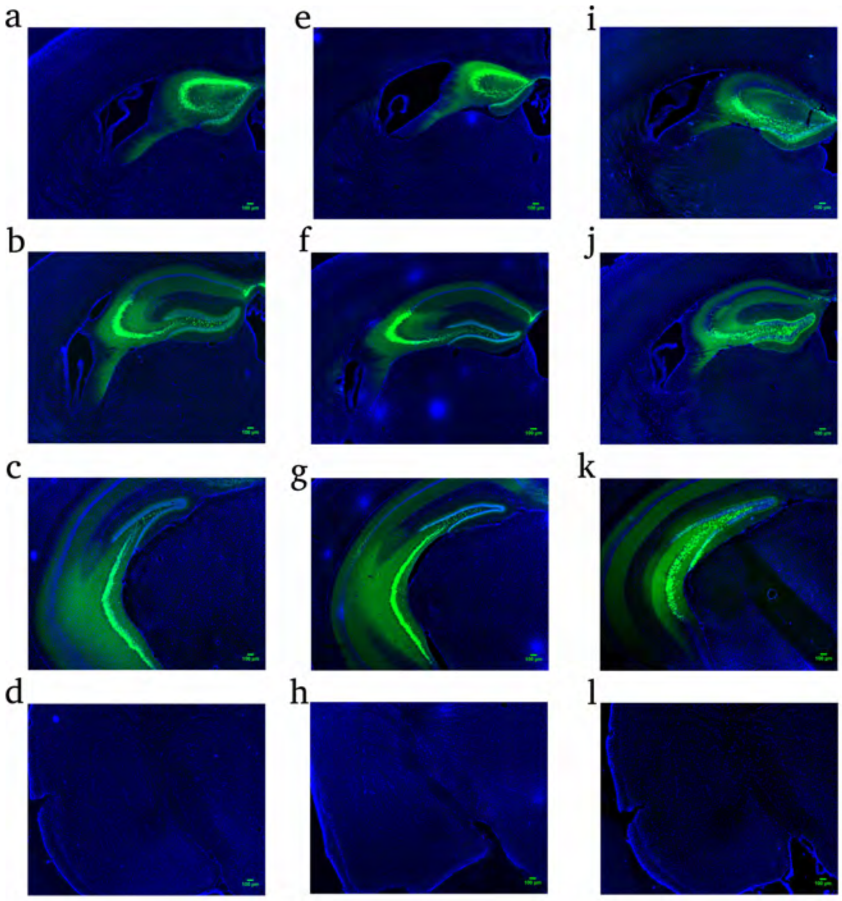

Figure S1. Detection of rAAV transfection on coronal brain sections based on fluorescent reporter expression. This figure shows pictures taken with a fluorescence microscope from coronal

sections of mouse brain injected with control, shCaMK2N1 or CaMK2N2 rAAV. Panels a to c show images of brain sections injected with control AAV, in an anterior-posterior order. Panel d shows a section of the same animal where the amygdalar nuclei can be seen. Panels e to g and panel h show images of brain sections treated with shCaMK2N1 rAAV. Panels i to k and panel l are images of animals injected with CaMK2N2 rAAV. All the pictures show DAPI nuclear staining in blue. The green colour in panels a to c and e to g has resulted from ZsGreen fluorescence, whereas in panels I to k it has resulted from eGFP fluorescence. Pictures were taken with a 2.5x objective and the scale can be seen on the bottom right corner of the images. Pictures confirm specific transfection and expression of rAAVs in the hippocampus.

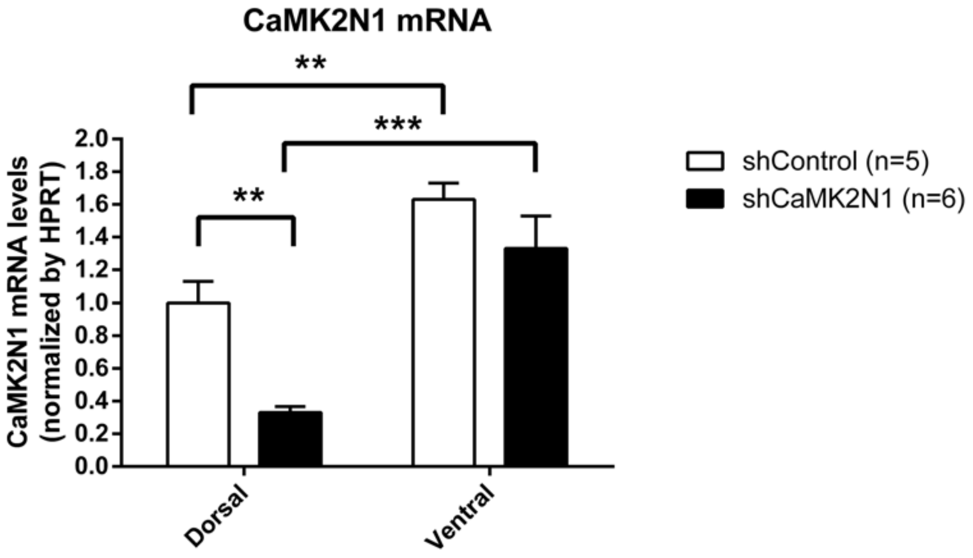

Figure S2. CaMK2N1 mRNA levels were reduced in dorsal, but not ventral hippocampus after shCaMK2N1 treatment. Quantitative analysis of CaMK2N1 mRNA levels in dorsal and ventral hippocampus of mice treated with either control (shControl) or shCaMK2N1-expressing rAAV. CaMK2N1 mRNA levels were normalized with the expression of the housekeeping gene HPRT. Two-way ANOVA test showed a significant effect of virus treatment ($F_{1,22}$=13.1; P=0.002) and a significant difference between hippocampal regions ($F_{1,22}$=37.1; P<0.001), with no interaction effect ($F_{1,22}$=1.9; P=0.18). SNK post-hoc comparisons showed that ventral hippocampus had higher levels of CaMK2N1 mRNA than dorsal hippocampus within the shControl group (q=4.5; P<0.01) and within shCaMK2N1 group (q=7.8; P<0.001). SNK test also indicated that the virus treatment resulted in a significant

decrease of mRNA levels of CaMK2N1 within dorsal hippocampus (q=5.0; P<0.01) but not ventral hippocampus (q=2.2; P=0.13). Based on this last result we have decided to work only with dorsal hippocampal samples. Means and S.E.M. are shown, ** P<0.01; *** P<0.001.

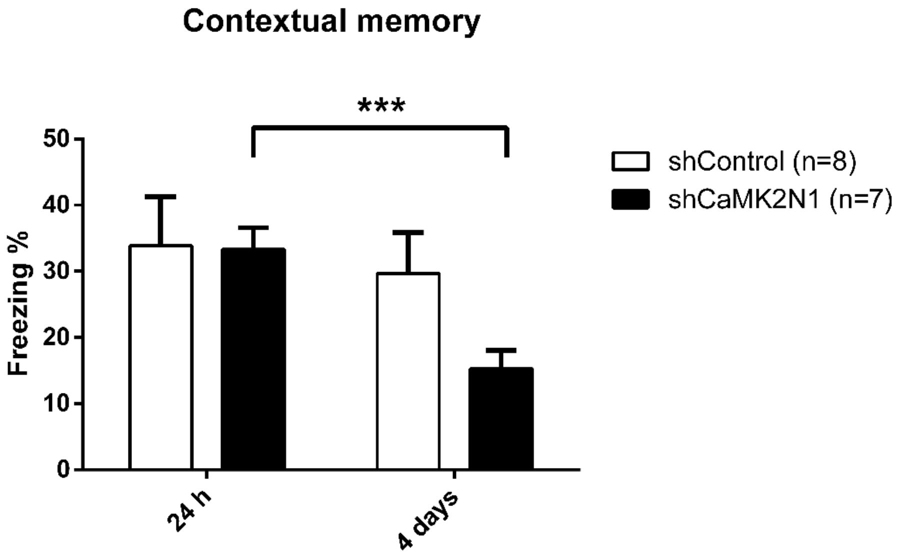

Figure S3. Independent experiment confirming that CaMK2N1 knockdown impairs LTM maintenance after retrieval. This figure shows the freezing scores obtained in a replica experiment with the training schedule as in Figure 1c. The replica experiment was executed by different researchers of our group. Two-way ANOVA test with repeated measures revealed no significant effect of treatment ($F_{1,13}$=1.3; P=0.271), but a significant effect of the memory tests ($F_{1,13}$=19.6; P<0.001) and a significant interaction effect ($F_{1,13}$=8.5; P<0.05). SNK post-hoc comparisons revealed a significant decrease in freezing scores between first and second memory test only for mice with CaMK2N1 knockdown (q=7.1; P<0.001). Means and S.E.M. are shown; ***P<0.001.

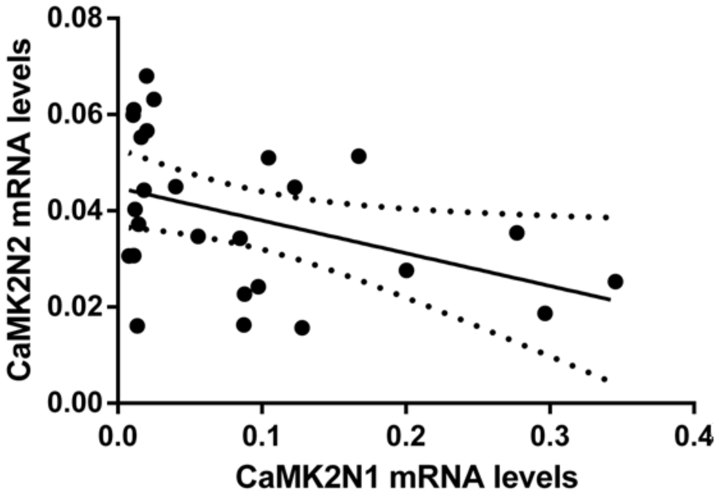

Figure S4 Linear regression between CaMK2N1 and CaMK2N2 mRNA levels. To test if the observed increase in CaMK2N2 (Figure 3c) was related to the decrease in CaMK2N1 (Figure 3b) we tested for a linear regression between CaMK2N1 mRNA and CaMK2N2 mRNA levels. Each animal is represented by an individual dot in the graph. Animals from both groups, shControl and shCaMK2N1, were included in the analyses. CaMK2N1 and CaMK2N2 mRNA levels were not standardized to any group. A significant negative linear regression was observed between the two variables (R^2=0.17; t=-2.24; ANOVA $F_{1,25}$=5.0; P<0.05), corroborating with the hypothesis that the increase CaMK2N2 mRNA levels might have occurred as a compensation to CaMK2N1 knockdown. Such a correlation was not observed when CaMK2N1 and CaMKN2 levels were analysed after only one LTM memory test (R^2=0.01; t=0.75; ANOVA $F_{1,38}$=0.5; P=0.45) (Figure 1).

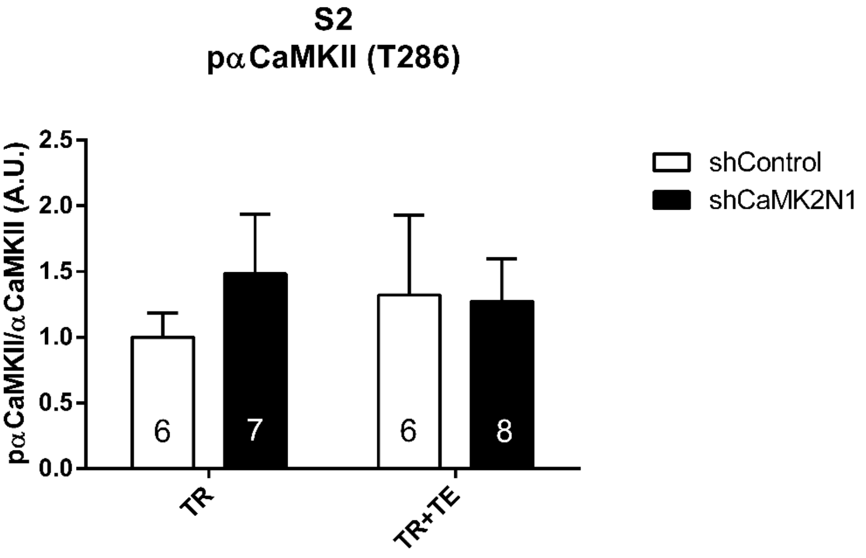

Figure S5. Retrieval does not change levels of αCaMKII autophosphorylation at T286 in cytosolic S2 fraction, and CaMK2N1 knockdown has no impact in this fraction. Levels of T286 phosphorylated αCaMKII (pαCaMKII) in the cytosolic fraction (S2) of animals trained (TR) or trained and tested (TR+TE) in the CFC paradigm. Animals of both groups received intra-hippocampal injection of either control (shControl) or shCaMK2N1-expressing rAAV. Levels of T286-phosphorylated αCaMKII were normalized by total αCaMKII levels. Two-way ANOVA revealed no significant effect for memory test ($F_{1,24}$=0.7; P=0.39), rAAV treatment ($F_{1,24}$=4.0; P=0.058) or interaction between these factors ($F_{1,24}$=0.4; P=0.51). N numbers for each group are represented inside the bars. Means and S.E.M. are shown.

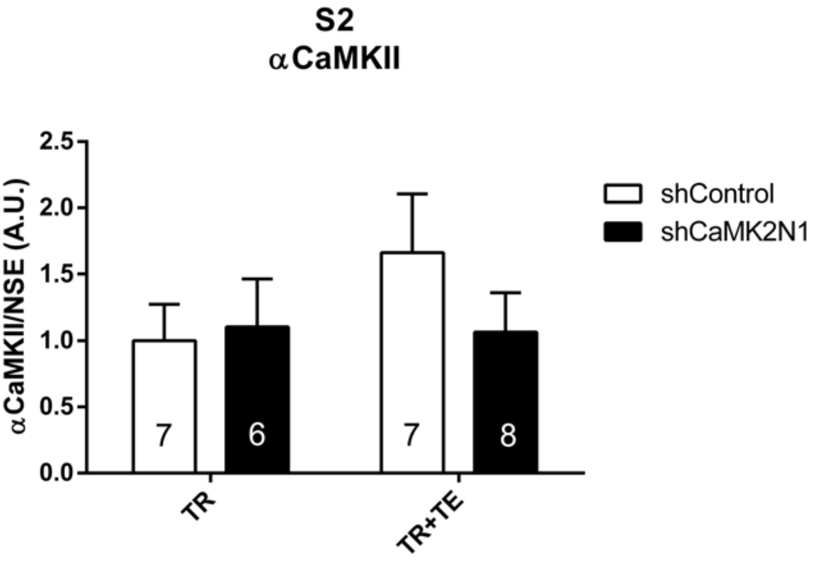

Figure S6. Total αCaMKII levels in cytosolic S2 fraction after CaMK2N1 knockdown. Western blots determined the levels of total αCaMKII in the cytosolic S2 fraction of animals trained (TR) or trained and tested (TR+TE) as in Supplementary Figure S5. Total levels of αCaMKII were normalized to neuron specific enolase (NSE) levels. Two-way ANOVA test showed no significant effect of memory test ($F_{1,26}$=1.1; P=0.30), virus treatment ($F_{1,26}$=2.2; P=0.15) or interaction between these factors ($F_{1,26}$=3.3; P=0.079). N numbers for each group are represented inside the bars. Means and S.E.M. are shown.

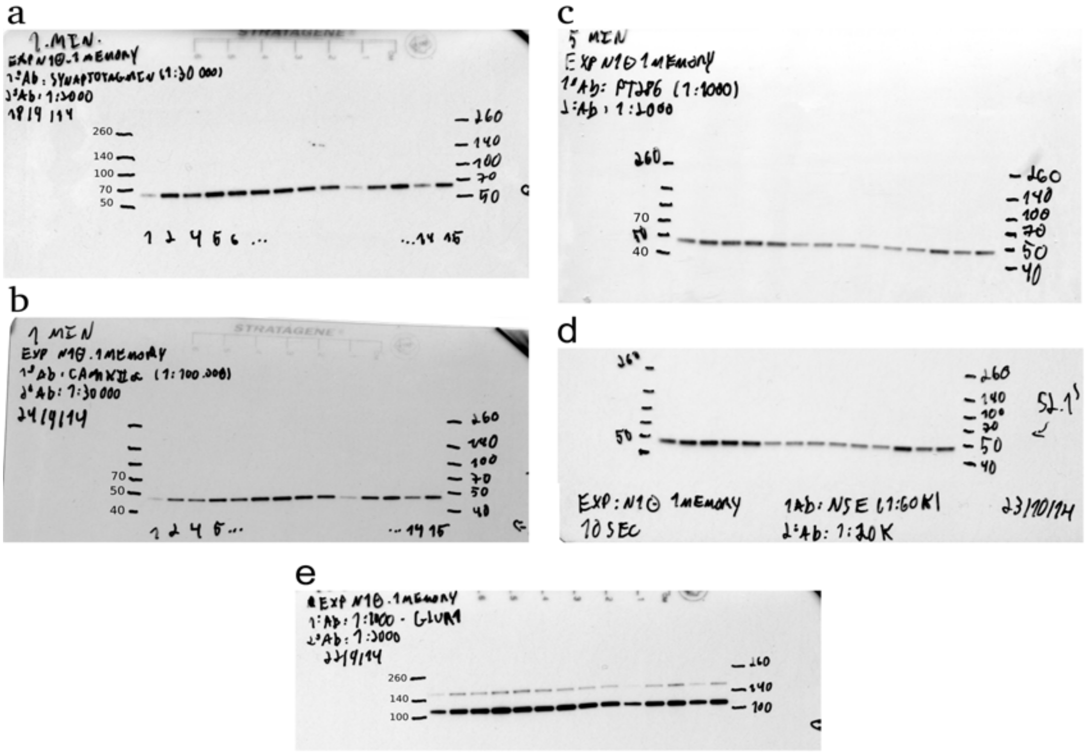

Figure S7. Representative western blot examples. Example western blots probed for synaptotagmin (a), αCaMKII (b), T286-autophosphorylated αCaMKII (c), NSE (d) and GluA1 (e).

Supplementary References

38 Drakulic, D. *et al.* Low-dose dexamethasone treatment promotes the pro-survival signalling pathway in the adult rat prefrontal cortex. *J Neuroendocrinol* **25**, 605-616, doi:10.1111/jne.12037 (2013).

39 Stanojlovic, M. *et al.* Repeated low-dose 17beta-estradiol treatment prevents activation of apoptotic signaling both in the synaptosomal and cellular fraction in rat prefrontal cortex following cerebral ischemia. *Neurochem Int* **83-84**, 1-8, doi:10.1016/j.neuint.2015.03.002 (2015).

40 Han, M. H. *et al.* Dysbindin-associated proteome in the p2 synaptosome fraction of mouse brain. *J Proteome Res* **13**, 4567-4580, doi:10.1021/pr500656z (2014).
